# Quality of Sexuality during Pregnancy, We Must Do Something—Survey Study

**DOI:** 10.3390/ijerph20020965

**Published:** 2023-01-05

**Authors:** Sonia García-Duarte, Bruno José Nievas-Soriano, Natalia Fischer-Suárez, Gracia Castro-Luna, Tesifón Parrón-Carreño, Gabriel Aguilera-Manrique

**Affiliations:** 1 Obstetrics and Gynaecology Unit, Torrecárdenas Hospital, 04009 Almería, Spain; 2 Nursing, Physiotherapy, and Medicine Department, University of Almería, 04120 Almería, Spain

**Keywords:** sexuality, pregnancy, females, sexual education, quality of life, sexual function, surveys and questionnaires

## Abstract

Background: The main aim of this study was to describe the changes in sexual desire in pregnant women during the gestation period. We also sought to analyze their sexual habits, how they perceive their partners’ attitudes, and determine if they know the importance of sex education during pregnancy. Methods: A cross-sectional descriptive observational study was conducted using an existing 32-item questionnaire among pregnant women attending the Fetal Welfare Clinic of a reference hospital. Univariate and bivariate analyses were performed. Results: One hundred seventeen women participated. 50.4% stated that their sexual interest decreased. Aspects like the number of coitus, feeling orgasm with penetration or with masturbation, or the frequency of oral sex, were significantly reduced during pregnancy. 90.6% of the women stated that they would like to receive more sex education during pregnancy. There were no differences according to their education level, their partners’ education level, or whether they believed that sexual intercourse could harm the baby. Conclusions: Several advances have been achieved in the healthcare that physicians and midwives provide pregnant women. However, the quality of women’s sex lives still declines during pregnancy. Healthcare providers should assume a proactive role, essential in helping future parents to enjoy their sexuality and dispel myths about sexuality, regardless of their education level.

## 1. Introduction

In 2000, the World Health Organization (WHO) defined sexuality as a fundamental dimension of being human [1]. Sexuality, understood as sexual life or sexual activity, is experienced and expressed in everything we are, feel, think and do [2]. It is a basic need that significantly impacts individual well-being and interpersonal relationships [3]. Thus, sexuality dramatically affects people’s quality of life and has historically and culturally oscillated between different degrees of recognition, from restriction to freedom [4]. However, its expression varies from one culture to another and according to the sociohistorical context [5]. Sexuality manifests differently depending on the stage of life [6]. The role of individual differences, learned factors, and sociocultural influences on women’s sexual responses are critical [7].

The enjoyment of sexuality should be adapted to the possibilities and desires of the couple [8]. However, sexual relationships go through many changes throughout a couple’s life, and the transition to parenthood can be considered a psychosocial life crisis [9]. Thus, one of the most critical stages in the life of a woman and her partner is pregnancy, where multiple and profound changes occur in the couple [9] due to fetal development and biological, social, and psychological adaptation to the state of gestation [10].

It has been described that, in the first trimester, hormonal changes cause a state of emotional lability. Many women also need to make a significant effort to maintain their usual sexual rhythm because of the appearance of nausea, vomiting, fatigue, and other discomforts [9,11]. However, in others, the opposite may occur [12]. Concerning orgasm in this trimester, in most women, the frequency and intensity seem to remain unchanged [11,13]. During the second trimester, many women report that sexual desire increases [12] because physical discomfort decreases or disappears and the couple has adapted to the pregnancy [10]. In the third trimester, most women experience a decrease or absence of sexual desire and a marked reduction of coital activity, often for fear of triggering labor [12,14]. The frequency and intensity of orgasm also seem to decrease, although the need for cuddling, caressing, and care seem to increase [14].

Multiple cultural prejudices transmitted throughout history have led to the pregnant woman being considered an asexual being only at the service of procreation, avoiding sexual relations [11]. Sexual intercourse during pregnancy was often taboo until not so long ago [15], as it was accepted that sex during pregnancy was dangerous, immoral, and indecent [13,15]. Even nowadays, women are overprotected. Oral and written tradition negatively influences sexual relations, imposing continence rules [15,16].

Thus, the cultural burden limits pregnant people’s sexuality, and health professionals should advise on sexual activity during pregnancy [17]. Some authors state that the consequences of sexual intercourse during pregnancy should be assessed individually, and the pros and cons of sexual activity should always be discussed with the pregnant woman [17,18]. Healthcare providers should incorporate specific counseling on sexual behavior for women and their partners, including discussing the impact of pregnancy [17,18,19]. Albeit there has been plenty of research about sexuality, historically, little importance has been given to sexuality in pregnancy [20], especially from physicians’ and midwives’ joint points of view. There are few studies about the sexuality of pregnant women, and even fewer ask the women how they feel about their partners.

Therefore, the main aim of this study was to describe the changes in sexual desire in pregnant women during the gestation period. We also sought to analyze their sexual habits and satisfaction regarding different sociodemographic, obstetric, and gynecological aspects, how pregnant women perceive their partners’ attitudes toward sexuality, and determine if women were aware of the importance of sex education during pregnancy.

## 2. Materials and Methods

### 2.1. Study Design

A cross-sectional descriptive observational study was conducted using an existing 32-item questionnaire developed to explore the main characteristics and modifications of sexuality in pregnancy and sexual education [21]. The exploratory questionnaire was defined by four domains: obstetric and gynecological aspects, sexual habits, degree of satisfaction with sexuality, and sexual education. As the questionnaire was exploratory, it did not require validation. The original questionnaire was developed initially in Spanish. Thus, it did not require translation or transcultural adaptation. An English translation of the questionnaire has been included as Supplementary Material.

### 2.2. Eligible Population, Sample Size Estimation, and Inclusion Criteria

The eligible population were pregnant women attending the Fetal Welfare Clinic (FWC) of the Hospital Torrecardenas (Almeria) for the first time. Almeria is a province located in the south of Spain with more than 720,000 inhabitants, and the Torrecardenas Hospital is the reference hospital for the entire province. In 2021, 3360 deliveries were attended at this hospital. Pregnant women are seen in the FWC from the 38th week of gestation. In 2021, 5040 consultations were performed for pregnant women in this office.

A maximum permissible error of 10% in estimating a proportion using an asymptotic normal confidence interval with correction for finite populations at 95% bilateral was considered for the sample size estimation. It was assumed that the proportion of respondents with decreased sexual interest during pregnancy was 45.10% [21] and that the total population size being 3360, it would be necessary to include 93 pregnant women in the study. Considering an expected dropout rate of 20%, it was required to recruit 117 pregnant women for the study.

The questionnaire was given to the pregnant women who attended the Fetal Well-Being Consultation at Hospital Torrecardenas for the first time and accepted to participate in the study. The inclusion criteria were: first-time visit to the Fetal Well-Being Consultation, being over 18 years of age, and speaking and reading Spanish. The exclusion criteria were: not meeting any inclusion criteria or not wishing to participate in the study. The questionnaire was self-administered by the participants independently.

### 2.3. Variables Analyzed and Statistical Analyses

The variables were divided into sociodemographic variables, sexual habits, degree of satisfaction with sexuality, and sexual education. The sociodemographic aspects of the pregnant woman and her partner collected were: age, educational level, and length of cohabitation. The age was categorized into the following strata to facilitate the analysis of specific aspects according to age: 18–19 years old, 20–23 years old, 24–27 years old, 28–31 years old, 32–35 years old, and >35 years old. The obstetric and gynecological variables were: contraception used, desired-planned pregnancy, number of pregnancies, number of abortions, life problems (psychological and personal conflict that produces important negative emotions or psychological discomfort), sexual problems, age at menarche, and age of onset of sexual relations. The variables on sexual habits were: sexual appetite during pregnancy, frequency of weekly coitus (before and during gestation), orgasm only with penetration (before and during gestation), orgasm with masturbation (before and during pregnancy), sexual appetite per week of the pregnant woman and her partner, sexual self-stimulation and frequency. The variables related to the degree of satisfaction with sexuality were: sexual satisfaction with the first coitus, situation of the first orgasm, the degree of attractiveness of the partner’s behavior during gestation, satisfactory sexual response, the importance of sexual relations, and harm of sexual relations to the baby. The variable on sexual education was: receiving more sexual education. Statistical analyses were performed using SPSS version 26 (IBM Inc., Armonk, NY, USA).

The statistical analyses were developed in three stages: univariate, describing frequencies and percentages for qualitative variables. For quantitative variables, means and standard deviation were used. For the bivariate analyses, Chi Square was used for qualitative variables with the appropriate corrections when the value in any cell was less than 5 (Linear-by-linear association). For nonparametric variables, the Wilcoxon test was used. Multivariate analysis was performed through binary logistic regression, taking the following as dependent variables for different models: desired pregnancy, planned pregnancy, and previous pregnancies, with several dependent variables with statistical or epidemiological significance.

### 2.4. Ethical Aspects and Review Board Approval

The study was conducted in accordance with the Declaration of Helsinki. The aims of the study were shown at the beginning of the questionnaire and informed consent was obtained. Personal data were not collected, and only fully completed questionnaires were admitted. The confidentiality of the participants was absolute as no personal data were collected or stored, and the researchers only could access completely anonymous questionnaires. The data collected was used without any statistical correction to perform the statistical analyses. All the data collected were managed according to the Spanish Law on the Protection of Personal Data (15/1999) and the Organic Law of Personal Data Protection and Warranty of Digital Rights (3/2018). This study was reviewed by the Research and Ethics Committee of the Nursing, Physiotherapy, and Medicine Department of the University of Almeria (Spain).

## 3. Results

### 3.1. Sociodemographic Aspects

One hundred seventeen women participated in the study. The mean age of the women was 31.2 years, and the mean age of their partners was 32.4 years. Significant differences were found when comparing the ages of pregnant women and their partners (Table 1). Regarding education level, 41% of the women had a university education, against 31% of their partners. These differences were also statistically significant (*p* < 0.001; Chi-Squared test, Linear-by-linear association).

Regarding the time they lived together, 72 (61.6%) women reported between 1 and 7 years, and 41 (35%) had been living together for more time (Table 2).

### 3.2. Obstetric and Gynecological Aspects

The mean age of menarche of the participants was 12.57 years. Sexual relations were initiated at an average age of 18.81 years, with a minimum of 13 years and a maximum of 31 years. 107 (91.5%) of the pregnant women reported that the pregnancy was desired, but only 76 (65.0%) stated it was planned. The mean number of pregnancies in our sample was 1.67, with a mean number of abortions of 0.41.

The most used contraceptive methods are shown in Table 3. The most used was the condom (34.8%), followed by oral contraceptives (29.6%). The least used ones were the injected contraceptives and intrauterine devices, both used by 0.9% of participants. No differences were found in the use of contraceptive methods regarding the age of the women, their education level, or the time of cohabitation (Chi-Squared test).

Twelve women (10.3%) stated having life problems. The most referred problems were sexual and relationship problems, referred to by five (4.3%) women, followed by personal and family problems, each stated by three (2.6%) women. One (0.9%) woman had vital problems because of pregnancy or delivery. A total of 48 (42.5%) women did not show health problems during pregnancy (Table 4). Among the ones that stated health problems, the most referred to were nausea or vomiting, noted by 41 (36.3%) women.

Women also were asked if they had experienced sexual problems during pregnancy. 46 (39.3%) of the total answered affirmatively. Twenty-eight (23.9%) reported a lack of desire, nine (7.7%) reported pain during intercourse, six (5.1%) reported genital infections, two (1.7%) reported an absence of orgasm, and one (0.9%) reported premature ejaculation in her partner.

### 3.3. Sexual Habits

Fifty-nine (50.4%) women reported that their sexual interest during pregnancy had decreased, 39 (33.3%) reported that it had remained the same, and 17 (14.5%) said that their sexual interest had increased. Regarding the number of coitus (Table 5), before pregnancy, the highest percentage was between 2–3 coitus per week (47%), but after becoming pregnant, most women answered it was one or less (64.1%). These differences were statistically significant.

Another item in the questionnaire was whether the women felt orgasm with penetration (Table 5). Before pregnancy, 67.5% of the women who answered this question felt orgasm always or almost always, dropping to 49.6% during pregnancy. 17.1% of the women rarely or never felt orgasm before pregnancy, rising to 33.3% during pregnancy. These differences were statistically significant.

The participants were also asked if they felt orgasm with masturbation (Table 5). Before pregnancy, 68.3% felt it always or almost always, dropping to 53.9% during pregnancy. 24.8% of women rarely or never felt orgasm before pregnancy, rising to 33.3% during pregnancy. These differences were statistically significant. However, no significant differences were found in these four sexual habits, during pregnancy, regarding the age of the women, their education level, the time of cohabitation, or the presence of health problems during pregnancy (Chi-Squared test).

Regarding oral sex, before pregnancy, 57 (48.7%) practiced oral sex sometimes or often, and this figure decreased to 34 (29.1%) during pregnancy (Table 5). These differences were statistically significant.

When asked about their sexual desire per week, 103 (88.1%) women answered between 1–4 times (Table 6), while 8 (6.8%) answered less than 1. No differences were found regarding education level, time of cohabitation, or health problems during pregnancy (Chi-Squared test, Linear-by-linear association). Regarding their husbands, 53 (45.3%) said their partner’s sexual appetite was higher than before pregnancy, 34 (29.1%) said their partner’s desire was the same, and 30 (25.6%) said their partner’s appetite was lower than before.

The last question of this domain was whether the pregnant women self-stimulated. 39 (33.3%) said they did, compared to 78 (66.7%) who did not.

### 3.4. Degree of Satisfaction with Sexuality

When asked if the women had felt satisfied with the first coitus, 65 (55.6%) said they had not felt pleasure. Regarding how they got their first orgasm (Table 7), 103 (88.1%) women got it through intercourse or masturbation, while 3 (2.6%) stated they had not had an orgasm yet. 82 (70.1%) reported having multiple orgasms.

When asked if they felt less attractive with pregnancy, 73 (62.4%) responded affirmatively. Regarding how the women felt their partner during pregnancy, 52 (44.4%) said they felt their partner was more passionate or affectionate than before. In comparison, 26 (22.2%) said their partner had less desire than before pregnancy (Table 7).

Regarding the satisfaction of their sexual relations, 99 (84.5%) women said they were satisfactory. When asked if they considered sexual relations important, 107 (91.4%) women considered them quite or very important, while 10 (8.6%) considered them a little or nothing important (Table 7). No differences were found in this aspect regarding their education level, the time of cohabitation, or the presence of health problems during pregnancy (Chi-Squared test, Linear-by-linear association).

### 3.5. Sexual Education

When the women were asked whether they would have liked to receive more sex education during pregnancy, 106 (90.6%) replied affirmatively. When asked if they believed that sexual intercourse could harm the baby, 103 (88%) answered negatively. No differences were found in these last two aspects regarding the women’s education level, their partners’ education level, the time of cohabitation, or the presence of health problems during pregnancy (Chi-Squared test).

Finally, no significant differences were found when they were asked whether they would have liked to receive more sex education during pregnancy regarding if they believed that sexual intercourse could harm the baby (Chi-Squared test).

The binary logistic regression analyses did not show any findings relevant to the study.

## 4. Discussion

The main aim of this study was to describe the changes in sexual desire in pregnant women during the gestation period. We also sought to analyze sexual habits and degree of satisfaction and determine if women were aware of the importance of receiving sex education during pregnancy.

### 4.1. Sociodemographic Aspects

As stated by other authors, the average maternity age has risen in the last decades [22], which seems to concur with our findings given the mean age of the women in our research. However, we must consider that women under 18 were excluded. We also found that few studies consider the age of their partners, which is often higher [23]. Regarding the education of the pregnant woman and her partner, we found higher education levels among women, an aspect that other authors have also found in their samples [23]. Albeit some researchers have assessed differences among married and cohabiting parents regarding pregnancy [24], few studies have assessed cohabitation regarding sexual aspects during this crucial period. In our study, most participants answered that they had lived together for between 4 and 7 years, which seems logical. In our environment, trying to get to know each other before deciding to have a child is understandable.

### 4.2. Obstetric and Gynecological Aspects

Although some authors have described that the most used contraception method is hormonal contraceptives [25], in our research, the method most used was the condom, as described by other authors [26]. Only a small percentage used the intrauterine device (IUD), an increasingly used method in younger women [27]. In our sample, less than two-thirds answered that the pregnancy was planned. The lack of pregnancy planning is one of the biggest reproductive and sexual health problems in all developed and developing countries [28]. However, framing unplanned pregnancy as a sexual health problem could also be controversial.

### 4.3. Sexual Habits

Other authors have pointed out that sexual interest decreases among pregnant women [29], something we found in our sample. Regarding specific sexual habits, our participants lowered the number of coitus per week during pregnancy, the number of orgasms with penetration and masturbation, and the frequency of oral sex, regardless of age, education level, time of cohabitation, or the presence of health problems during pregnancy. These are critical findings. As far as we know, no other studies have described differences in the number of orgasms like the data in our research, among women who felt them before being pregnant and those who did not feel them. Several advances have been achieved in the healthcare that physicians and midwives provide pregnant women. However, the quality of women’s sexuality declines when they become pregnant. We agree that cultural factors may diminish the sexual interest of the pregnant woman [29,30,31] since, until not long ago, pregnant women were considered asexual beings [11,32]. Psychological factors are also crucial since the pregnant woman tries to protect the fetus and does not know if sexual relations may harm the future baby [33]. In summary, the results of our study show that even nowadays, the quality of women’s sexual life is still burdened by social, psychological, and cultural factors that limit their potential and thus impoverish their overall quality of life.

### 4.4. Degree of Satisfaction with Sexuality

One possible explanation for the pregnant woman’s loss of sexual interest is that she sees herself as less attractive [32]. Our findings agree with this, as almost two-thirds reported seeing themselves as less attractive during pregnancy. This view may be due to the changes inherent in pregnancy. Several authors have described that these reasons can affect a woman’s attractiveness and how she perceives herself [32]. In this connection, some authors have described that women’s sexual desire decreased during pregnancy, and some men also reflected this [29]. However, in our study, many women answered that their partners’ sexual desire increased.

### 4.5. Sexual Education

As stated by other authors [34,35], sex education is essential at all stages of life, especially during pregnancy. When the pregnant women were asked whether they would have liked to receive more information on sex education during pregnancy, most answered affirmatively. Most women responded negatively when asked if they believed that sexual intercourse could harm the baby. These answers seem logical. However, no significant differences were found when they were analyzed regarding the women’s education level or their partners’ education level. Moreover, when analyzing whether they would have liked to receive more information on sex education during pregnancy and if they believed that sexual intercourse could harm the baby, significant differences were not found.

These are also critical findings: the educational level of both women and their partners makes no difference in being aware of whether more sex education would be helpful or whether sex during pregnancy can harm the baby. Even more, believing whether or not sex harms the fetus also does not differentiate women who believe they could benefit from more pregnancy-related sex education. Therefore, as stated by other authors [36,37], healthcare providers should assume a much more proactive role, essential in helping future parents enjoy their sexuality, and dispel myths about sexuality, regardless of their education level.

### 4.6. Limitations and Strengths

The most important limitation of this study is the potential selection bias, as the eligible population was from a specific hospital consultation. Although the hospital was of reference for a population higher than 720,000 inhabitants and the consultation was ideal for recruiting women in the final stages of their pregnancy, this aspect should be considered when assessing our conclusions’ external validity. As this is a self-report study, participants may have exaggerated, underreported, or misremembered their answers. Another limitation of the study is that it was necessary to be able to speak and read Spanish to participate. Therefore we have not considered the migrant women who attended the fetal well-being consultation but could not read or speak Spanish fluently. Another selection bias of the study comes from the participation age since, to participate, the women had to be 18 years or older. We must also consider that we did not retrieve the opinion of the participants’ partners, as we obtained these data through the subjective vision of the pregnant women. As only one question referred to the body image of pregnant women, this aspect may be underexplored, and future research can deepen this aspect. Finally, the presence of health problems during pregnancy may be a contraindication to intercourse during pregnancy. Thus, this aspect must be considered when assessing the results.

This study also has some strengths. The sample size was adequate, according to the sample estimation previously performed. One of the most important strengths is that the sample consisted of real women obtained from a reference hospital. Thus, their answers reflect the current perception of these women about this important topic. This aspect allows us to obtain conclusions that can be helpful for other researchers, even if they may need to be assessed prudently.

For future research, it would be helpful to perform this study in other provinces or countries or to include a larger number of participants. It would also be beneficial to use a separate questionnaire to obtain answers directly from the partners. Another future project could be carried out with pregnant women attending maternal education and providing a section on sexual education. In this way, we could study if sexual education directly affects the sexuality of pregnant women. It also could be helpful to analyze multiparous women separately from primiparous women. Thus, it would be possible to investigate whether the previous pregnancy experience affects how they relate to their partner. Future studies could also focus on obtaining and analyzing more partner data.

## 5. Conclusions

Several advances have been achieved in the health cares that physicians and midwives provide pregnant women. However, the quality of women’s sex lives still declines when they become pregnant. Albeit the women can feel their partners more passionate at this stage, they also feel less attractive. Significant differences can be found in the frequency of feeling orgasms among pregnant women who felt them previous to being pregnant and those who did not feel them. Another critical finding is that the educational level of both women and their partners, or whether believing or not that sex harms the fetus, makes no difference in being aware of whether more sex education would be helpful for them. Therefore, healthcare providers should assume a much more proactive role, essential in helping future parents to enjoy their sexuality and dispel myths about sexuality, regardless of their education level.

## Figures and Tables

**Table 1:** Sociodemographic variables of the pregnant women and their partners.

Age	Mean	SD	*p*-Value *	
Pregnant women	31.2	5.03	0.001	
Partners	32.5	5.15		
Education level	Pregnant women		Partners	
	n	%	n	%
Primary	36	30.8	37	31.9
Secondary	13	11.1	14	12.1
University	48	41.0	36	31.0
Other studies	20	17.1	29	25.0
Total answers	117	100.0	116	100.0

* Wilcoxon’s test.

**Table 2:** Time of cohabitation.

	n	%
Less than one year	4	3.4
1–3 years	27	23.1
4–7 years	45	38.5
8–11 years	22	18.8
More than 11 years	19	16.2
Total answers	117	100.0

**Table 3:** Contraception used.

	n	%
Condom	42	35.9
Oral contraceptives	35	29.9
Reversal (coitus interruptus)	19	16.2
No contraceptive methods	19	16.2
Injectable contraceptives	1	0.9
Intrauterine device (IUD)	1	0.9
Total answers	117	100.0

**Table 4:** Health problems during pregnancy.

	n	%
No problems	49	41.9
Nausea or vomiting	42	35.9
Diabetes	10	8.5
Uterine contractions	7	6.0
Metrorrhagia	2	1.7
Other	5	4.3
Total answers	117	100.0

**Table 5:** Sexual habits.

	Before Pregnancy		During Pregnancy		*p*-Value
Coitus per week	n	%	n	%	
More than three	32	27.4	9	7.7	<0.001 ^a^
Two or three	55	47.0	33	28.2	
One or less	30	25.6	75	64.1	
Total	117	100.0	117	100.0	
Feel orgasm with penetration	n	%	n	%	
Always	41	35.0	31	26.5	<0.001 ^b^
Almost always	28	32.5	27	23.1	
Half of the times	18	15.4	20	17.1	
Rarely	16	13.7	20	17.1	
Never	4	3.4	19	16.2	
Total	117	100.0	117	100.0	
Feel orgasm with masturbation	n	%	n	%	
Always	61	52.1	47	40.2	<0.001 ^a^
Almost always	19	16.2	16	13.7	
Half of the times	8	6.8	15	12.8	
Rarely	14	12.0	19	16.2	
Never	15	12.8	20	17.1	
Total	117	100.0	117	100.0	
Oral sex	n	%	n	%	
Often	11	9.4	3	2.6	<0.001 ^a^
Sometimes	46	39.3	31	26.5	
Rarely	24	20.5	38	32.5	
Never	36	30.8	45	38.5	
Total	117	100.0	117	100.0	

^a^ Chi-Squared test, ^b^ Linear-by-linear association.

**Table 6:** Sexual desire per week.

	n	%
Less than 1 per week	8	6.8
1–2 times a week	70	59.8
3–4 times a week	33	28.3
5–6 times a week	4	3.4
Seven or more	2	1.7
Total	117	100.0

**Table 7:** Degree of satisfaction with sexuality.

How They Got Her First Orgasm	n	%
Intercourse	58	49.6
Masturbation	45	38.5
Sexual fantasies	7	6.0
Not had an orgasm yet	3	2.6
Total	117	100.0
How do they feel their partner	n	%
More passionate	10	8.5
More affectionate	42	35.9
Same as before	39	33.3
Has less desire	26	22.2
Total	117	100.0
Importance of sexual relations	n	%
Very important	48	41.0
Quite important	59	50.4
A little important	7	6.0
Nothing important	3	2.6
Total	117	100.0

## Data Availability

Not applicable.

## Supplementary Materials

The following supporting information can be downloaded at: https://www.mdpi.com/article/10.3390/ijerph20020965/s1.
