# Supplementary material for: Quality of Sexuality during Pregnancy, We Must Do Something—Survey Study
Source: Int J Environ Res Public Health. 2023 Jan 5;20(2):965. doi: 10.3390/ijerph20020965 (PMC9859225; doi:10.3390/ijerph20020965)
Supplement: Supplementary file 1 [file ijerph-20-00965-s001.zip › ijerph-1928868-supplementary.pdf]

Quality of sexuality during pregnancy, we must do something — Survey Study

Supplementary material: English translation of the exploratory questionnaire

Part 1. Sociodemographic aspects

1. What is your age?
   a) 18-19 years
   b) 20-23 years
   c) 24-27 years
   d) 28-31 years
   e) 31-35 years
   f) Older than 35

2. Age of your partner
   a) 18-19 years
   b) 20-23 years
   c) 24-27 years
   d) 28-31 years
   e) 31-35 years
   f) Older than 35

3. Your education level is:
   a) Primary
   b) Secondary
   c) University
   d) Other studies

4. Your partner's education level is:
   a) Primary
   b) Secondary
   c) University
   d) Other studies

5. The time of cohabitation with your current partner is:
   a) Less than one year
   b) 1-3 years
   c) 4-7 years
   d) 8-11 years
   e) More than 11 years

Part 2. Obstetric and gynecological aspects

1. Age of menarche: (years)

2. Sexual relations were initiated at: (years)

3. The current pregnancy was desired:
   a) Yes
   b) No

4. The current pregnancy was planned:
   a) Yes
   b) No

5. Previous number of pregnancies: (number)

6. Previous number of abortions: (number)

7. Contraception method used preferably:
   a) No contraceptive methods
   b) Condom
   c) Oral contraceptives
   d) Reversal (coitus interruptus)
   e) Injectable contraceptives
   f) Intrauterine device (IUD)

8. Do you have any vital problems?
   a) No vital problems
   b) Sexual and relationship problems
   c) Personal and family problems
   d) Pregnancy or delivery problems

9. Health problems during pregnancy:
   a) No health problems during pregnancy
   b) Nausea or vomiting
   c) Diabetes
   d) Uterine contractions
   e) Metrorrhagia
   f) Other

10. Sexual problems during pregnancy:
   a) None
   b) Lack of desire
   c) Pain during intercourse
   d) Genital infections
   e) Absence of orgasm
   f) Premature ejaculation in her partner

Part 3. Sexual habits

1. Sexual interest during pregnancy:
   a) Decreased
   b) Remained the same
   c) Increased

2. Number of coitus before pregnancy
   a) More than three
   b) Two or three
   c) One or less

3. Number of coitus during pregnancy
   a) More than three
   b) Two or three
   c) One or less

3. Orgasm with penetration before pregnancy:
   a) Always
   b) Almost always
   c) Half of the times
   d) Rarely
   e) Never

4. Orgasm with penetration during pregnancy:
   a) Always
   b) Almost always
   c) Half of the times
   d) Rarely
   e) Never

5. Orgasm with masturbation before pregnancy:
   a) Always
   b) Almost always
   c) Half of the times
   d) Rarely
   e) Never

6. Orgasm with masturbation during pregnancy:
   a) Always
   b) Almost always
   c) Half of the times
   d) Rarely
   e) Never

7. Practice oral sex before pregnancy:
   a) Often
   b) Sometimes
   c) Rarely
   d) Never

8. Practice of oral sex during pregnancy:
   a) Often
   b) Sometimes
   c) Rarely
   d) Never

9. Current sexual desire per week:
   a) Less than 1 per week
   b) 1-2 times a week
   c) 3-4 times a week
   d) 5-6 times a week
   e) Seven or more

10. Current partner's sexual appetite:
   a) Higher
   b) The same
   c) Lower

11. Current self-stimulation
   a) Yes
   b) No

Part 4. Degree of satisfaction with sexuality

1. You felt satisfied with your first coitus
   a) Yes
   b) No

2. You got their first orgasm with:
   a) Intercourse
   b) Masturbation
   c) Sexual fantasies
   d) Not had an orgasm yet

3. You feel less attractive during pregnancy:
   a) Yes
   b) No

4. During pregnancy, you feel your partner:
   a) More passionate
   b) More affectionate
   c) Same as before
   d) Has less desire

5. Importance of sexual relations in your life:
   a) Very important
   b) Quite important
   c) A little important
   d) Nothing important

6. Your sexual relations are:
   a) Satisfactory
   b) Not satisfactory

Part 5. Sexual education

1. You would like to receive more sex education during pregnancy:
   a) Yes
   b) No

2. You believe that sexual intercourse could harm the baby:
   a) Yes
   b) No
